# Animal Chemistry and Its Relation to Therapeutics

**Published:** 1860-05

**Authors:** J. L. Teed

**Affiliations:** Mendota, Ill.


*Animal Chemistry and its Relation to Therapeutics.*—By J. L. Teed, M. D., of Mendota, Ill.

In nothing is the science of the present day so different from that of all former periods with which we are acquainted as in its utilitarian tendencies, and in its requirement of practical demonstration; and this tendency has been gradually advancing in proportion as it has demonstrated the superiority of a foundation established on fact, to one resting merely on theory or hypothesis. The acute mind of Magendie was long since convinced of the insufficiency of the latter, even although propounded by a Bichat, and of the absolute necessity that physiology should be the offspring of experiment alone, and physiology soon found in animal chemistry the only true exponent of its phenomena. The chair of experimental physiology in Paris is the result of that conviction, and M. Magendie's pupil, M. Claude Bernard, is giving fresh demonstrations of the correctness of these conclusions. The researches of Mr. Brown-Sequard are further proofs of the same truth; and the doctrines elaborated by the researches of these, and others in the same direction, are as much the property of animal Chemistry, as that the forces which operate on inorganic matter belong to the general chemist. While animal chemistry embraces the nature and composition of the animal solids and fluids, it includes also the laws and sources of their formation and the forces by which they are regulated and maintained or removed beyond the sphere of the organism.

The researches of the German schools, however, have perhaps been productive of the greatest results. Among these it would be invidious to particularize, with the exception of one, who may be said to be the father of this branch of science, and the name of Justus von Liebig should be a household word to the student of nature, whether of the animal, vegetable, or mineral kingdoms.

To his individual mind animal chemistry owes much—very much; and to his teachings and example, and to the direction which he gave to the minds of so many others, a very large proportion of our present knowledge is due. Deduct the teachings of the last twenty-five years contributed by Liebig and his pupils, and there remains but little of animal chemistry over what was known twenty-five years ago. If, also, the advance of science in the past quarter of a century has been so great from a beginning so numerically small, what may be expected as the result of the investigations of the next quarter of a century, with its ever-increasing army of investigators; then it was no very difficult thing to keep pace with the general improvement, now the fresh researches in one branch alone constitute a study of themselves, whilst the whole are absolutely necessary to enable the physician to do his duty to his patient and justice to the profession of which he is a member.

We must not forget, however, that the science is yet too little advanced in the details of all its branches to furnish us with that sure and unerring light which leaves the physician nothing further desirable to guide him in the mysterious paths of therapeutics, and to enable him at all times to combat successfully the ravages of disease. Nor must we hope that this result will ever be gained. To avert death from a mortal is a simple absurdity; fit, indeed for the boastings of a Paracelsus, who died with his "elixir vitæ" in his pocket, a monument of folly; but to postpone the event of dissolution twixt soul and body; to cut short in their commencement those series of morbid actions and changes, which, if continued, would terminate in death, so that this last may be the result only of the laws of being, or, where this is impossible, to smooth the passage to the grave, are the great ends which the physician proposes to accomplish, and the decreasing mortality of many diseases, and the increasing duration of human life, testify to the advancement of scientific or rational medicine.

Now, in what does the rational physician differ from the empiric? The latter uses a remedy or a course of treatment, because in such cases it has been found serviceable. Experience has been his guide; and to this we do not object, when in any case we have reached the limits of scientific or rational knowledge. This has been the discovery and ground of introduction, and consequent use or disuse of nearly all the articles of our materia medica; nor do we propose to throw away this guide in the treatment of disease, until we have a better to substitute for it.

But when experience has recommended a certain course of treatment in any particular disease, what shall we say of the unsuccessful cases? They are not recommendations of the treatment; but they must not be thrown aside. The experience derived from them may furnish no indications for the next case; no ground for an alteration of the treatment; and to treat the next case in the same way, is to fly in the very face of that principle vaunted as a guide. The empiric, however, has nothing left but to pursue the same course. In all such cases, and every practitioner of medicine and surgery knows their frequency, bringing with their recollections feelings of sadness and vain regrets—in all such cases, a plan of investigation which promises to throw a gleam of light across the darkness, should be hailed with delight and welcome; and the well-balanced mind will avoid the two evils of expecting too much from it on one hand, and rejecting a valuable auxiliary on the other.

The rational physician will, therefore, use experience as his guide, when he has no better; but he will at the same time endeavour to extend his knowledge of the modus operandi of remedial agents, of the nature and properties of the organism, and of the laws governing it in its whole and in its varied parts, and of the variations induced by external or internal causes. By these means experience, when correct, will be confirmed; when erroneous, will be corrected.

As a whole, therefore, and comprising all these branches of research, animal chemistry presents itself to the rational physician offering him a reliable guide in all the cases over which its investigations have been carried; and if these be yet few, their importance demands attention to the subject, and is the promise of a future and more abundant harvest.

Animal chemistry, as has been before remarked, is so very closely allied to animal physiology, that the latter may be said to be embraced, at least to a very considerable extent, in the former; in the same way as the forces which cause changes in inorganic matter belong to inorganic chemistry, the fact of these forces being more numerous, more complex, and less easily investigated, is no sufficient ground for their exclusion.

The branches, therefore, belonging to animal chemistry are that portion of embryology which treats of the primary constitution of the embryo and its appendages; the period and manner of appearance of fresh elements in it; the successive steps in its formation, manner of growth, sources of nutrition, and internal changes; distribution of effete matter, if any; modifications from abnormal or other causes, producing alterations of development, disease, or death. In this department of the subject very little is yet fully made out, as regards the chemical changes and modifications undergone by matter while it is being converted into the perfect embryo; these, however, are most important, and are receiving a large share of investigation.

The chemistry of the tissues is another and an important division of this subject, and one which hardly needs more than mention, as it must be evident that the most minute acquaintance with the composition of any body, in its whole and in its parts, is a necessary prerequisite to its complete study.

The metamorphosis of tissue is so intimately connected with two other divisions, viz., the chemistry of food and the chemistry of the excretions, and when to these have been added the chemistry of the secretions and of nutrition, the importance of the subject looms up before the mind in its vastness and sublimity; the force of the nervous system capping the whole, and impressing the reflective mind with its unspeakable grandeur.

Follow on, then, the mutations from ordinary causes which constitute disease, the effects of extraordinary ingesta, *i. e.*, of those not food, such as of medicines and poisons, from cold water to the most active and deadly matters, and a subject is mapped out having no parallel amongst material things in its importance to the welfare of humanity.

A great difficulty in the outset of this subject, which presents itself to the investigator, as he will find the matter treated of by various authors, is the assumption of "means" as standards—take, for instance, the composition of the blood: one analysis is made, giving so many parts by weight as the amount of water; another is made, giving a result somewhat different, and so on with the other constituents—the mean is then calculated for each constituent, and received as the proportional quantity for such constituent in the condition of health, or of any one disease, as the case may be, and so on through the secretions and excretions, &c. There is a very important reason why this method of investigation is injurious, and even fallacious.

We will allow that the composition of the body, or of any part of it, does vary in different individuals; yet in every individual there are four points which must be complementary of one another to make up the whole, and have therefore fixed relations to eath other: these are first the composition of the body itself, fluids and solids; the composition of the food, that of the excretions, and the metamorphosis of tissue in nutrition and secretion. The weight of the body taken at any certain time, say January 1st, plus the weight of food and drink taken, and air, inspired up to say February 1st, must equal the weight of the body on February 1st plus the weight of the excretions from January 1st to February 1st. It is also found that any alteration in diet will produce a corresponding alteration in the constitution of the excretions; it must, therefore, produce a corresponding alteration in all the parts lying between the food, digestion, and the excretions; nor should we, *a priori*, expect to find the *tout ensemble* of a man living on a succulent vegetable and fruit diet, cereals and animal matters, milk, eggs, &c., &c., being only very sparingly used, or entirely abstained from, the same as in the case of another man living almost or altogether on cereals and flesh. The particular diseases to which the followers of certain dietaries, whether from necessity or choice, are subjected, or from which they are exempt, are striking illustrations of the same fact. Connect the Icelander feeding on blubber with his freedom from phthisis, and liability to diseases of the skin and bowels; the eaters of maize in hot countries, with the liability to skin disease; the liver on salt food, the prevalence of scurvy; the butcher and publican, and an immunity from phthisis; and so on through examples which must occur to all, and the intimate material connection between these four points become strikingly evident.

If such then be the fact, it is evident that in any given case the chemical constitution of the blood requires an acquaintance with the food out of which that blood has been formed, and also of the execretions during the time, by which its formation has been regulated, to say nothing of the particular growth of this or that tissue.

Again, as there are four excretions—those of the lungs, kidneys, bowels and skin—and as these are complementary of one another, according to the activity of each, varying continually in the same individual, even while using a diet the same both in quantity and quality, according to the ever-varying impressions of external causes, such as rest, motion, heat, cold, &c., as also from internal causes of extreme mobility, it will necessarily follow, not that urine or sweat, or expiration or feces, have a variable composition, but they have a composition varying according to the complementary variations of each particular case, *i. e.*, that their composition is fixed, the circumstances of their formation being the same; and, in order that the analysis of either excretion may be made of much value, those of the other three, of the food from which they have been derived, and of the blood or medium of formation also, should be given.

The main object in the study of animal chemistry should be to reach the laws under the operation of which the various changes in the body occur first in those of more general application; and then, as the modifying circumstances are ascertained, those of more limited or special range. The special composition of such an excretion, of such a fluid, of such a solid, is of far less consequence than the full knowledge and comprehension of the circumstances under which it was formed, and of the laws which regulated the due and proper fulfilment of those functions of which the substance in question, whatever it may be, is the result.

For instance, take the urinary secretion; suppose we know its normal composition most accurately, and on examining a sample we find an alteration in some most important particular; thus let the urine under examination contain a large amount of sugar. The first question which arises is, What are the laws governing the formation of sugar in the economy? Where is it formed? From what is it formed? What are the residuary or complementary products of its formation, or is it itself a residuary product? Whence are its derivatives obtained? What should be the proper quantity formed, if any? What is its normal destination and the purpose it is intended to fulfil? Is its appearance in the urine the result of its non-disappearance elsewhere, or the result of excessive formation, or the result of previous retention? &c., &c. Each one of these questions, though most physiological in itself, requires a chemical investigation to supply the answer. The same course of inquiry reaches far and wide through the whole course of therapeutics; and let any one consider these questions carefully, then look at the empirical treatment of diabetes mellitus, with the usual termination of the case, and answer to himself, How often would the result be different if these questions could be satisfactorily answered? This example is a striking and prominent one; but many are the cases in which a large amount of suffering might be relieved more effectually than at present, were the knowledge of this subject more extended.

The chemistry of food and drink is a very important branch of this subject. From the milk at its mother's breast to the multifarious diet of mature life has to be manufactured a constant stream of nutritive element, supplying the various tissues with their means of nutrition and growth. The processes by which these articles of food are converted into blood are of the first order of importance, and closely connected with them are the means by which superfluous or otherwise disorderly elements are removed. The body may be said to be made up of certain elements, C,O,H,N,I,P,Cl,K,Na,Fe, and some few others of less importance, in varying proportions, the matters excreted from it are composed of the same ingredients in more definite and fixed combinations. The food must, therefore, have the same elementary composition, and in the same proportions, as the excreted and retained matters; and any other element, if foreign, or any one of these, if superfluous, must either be rejected, or cause a greater or less alteration if retained. If rejected, it may undergo decompositions, fresh groupings of elementary atoms occur, and the processes of life suffer derangement from the presence of foreign bodies.

In health, therefore, it is important to know the composition of the food consumed, in order to be enabled to calculate on a harmonious working of the whole system, in its varied parts of digestion, nutrition and secretion, and to be able to supply any particular emergency which may arise from extraordinary circumstances. But of how much more consequence is this in disease. How important to know how much and what kind of nourishment should be snpplied to meet the wants of the system and leave no surplus, the nidus of further mischief. How important to be able to adjust the kind of food supplied to the assimilating powers of the invalid. How often in chronic cases do we find that a depraved blood stream is the cause of the evil, having its *Fons et origo* in some mal-adjustment between the food taken and the processes of assimilation; when, having gently increased the excretions for a short time, so as to remove any accumulation of peccant or ill-assimilated matter, we slightly shorten the supply of food, modulate its quality according to the circumstances of the case, use some gentle alterative, and find our attentions rewarded by the grateful patient becoming a confiding friend? Were this subject more investigated, and better understood, the public would never turn homœopathic, to avoid being drenched *usque ad nauseam*, and patients would soon understand that the advice of a physician is often of more value than his medicine.

The changes undergone by the food in digestion have been subjected to much investigation, and considerable light has been thrown upon them. Indeed, on reading some authors, it might be supposed that our knowledge of this subject was complete, or nearly so. This, however, is by no means the case; and the changes induced in nearly all kinds of food, before they are converted into serum and corpuscles, are but little more than conjectures. The difficulty attending the whole investigation is so immense, owing chiefly to the extreme mutability of the matters formed, and the impossibility of obtaining them in an isolated state, by our present methods of analysis; our inability at present to measure the effects of nervous influence on the production of healthy or morbid transitions, together with our inappreciation of slight disturbing causes, that our knowledge is yet but little more than inceptive. We have, however, this encouragement, that if so much may be done with so little knowledge, what may we expect when our acquaintance with the subject is more extended, and what inducements does it hold out for its assiduous cultivation.

The relations of animal chemistry to nutrition and growth is most intimate and important; the body increasing in weight, in different proportions in its different parts, requires an attentive consideration; while in mature life the due adjustment of its varied constituents is necessary to full and continued vigor, with a strong resisting power against disturbing influences. Errors in nutrition lie at the root of many diseases, and the most successful treatment of one of the most destructive, scrofula in its varied forms, is a treatment almost entirely based on this foundation, with reference to assimilation and diet.

The chemistry of the excretions has received the most attention, chiefly, perhaps, from these being so very sensibly altered in disease, and the readiness with which they have been received as exponents of the morbid changes going on in the system. Without wishing to depreciate their actual importance, they have received more than their relative share of consideration. They are most certainly intensely altered in morbid conditions of the system, and do afford most valuable indications of such conditions. But the idea of combining a certain state of any particular excretion with a particular condition of morbid action, and making that as the entire basis for treatment, has often led to most disastrous results, by withdrawing the attention of the physician from the system to a symptom, whereby the disease has continued its course unchecked—fortunately for the patient if unaggravated by the treatment employed. Snch an occurrence could never happen if all the outlets of excretion were attentively examined, and the compensatiug relations of the excretions themselves considered; then attention being paid to the parts retained as well as to those discharged, and to the sources from which these all are derived, would put the physician in possession of all the facts of the case, and lead him to a correct conclusion, both as to the nature of the malady and its treatment.

Diseases may be considered as grouped into certain families: thus, we may enumerate pyrexia or fevers; phlegmasia or inflammations; neuroses, or those which have an origin or seat chiefly in the nervous system. Besides which there are certain diseases of the chylopoietic viscera and skin, not, perhaps, capable of being classified under either.

In the case of the pyrexiæ the utmost importance attaches to a correct understanding of the chemical changes which should occur in health, and which do happen in the disease, and to the influence of remedial agents in preventing, counteracting, and removing those morbid products which keep up the disease, and by their presence and decomposing influences induce still further disorganization. The treatment of fevers is essentially a chemical process. We use evacuants to remove accumulations of effete matters, and thereby purify the residue; we act on one organ to relieve the system of matters which should have been discharged by another; we adjust our supplies of nourishment to the capabilities of the system to assimilate; we supply stimulants to maintain the nerve-force under depression; in short, all our proceedings are just as chemical as the mixing an ore with a flux to obtain the metal in a pure and separate state; or as the proceedings of the artist to produce his photograph or his daguerreotype. We examine the secretions with the view of finding their chemical constitution, and the knowledge of the chemical changes induced by remedial agents enables us to understand the success which follows seemingly opposite modes of treatment; the better we are acquainted with animal chemistry, with the greater facility shall we be enabled to adjust our treatment to the exigencies of the case, and the greater will be the success attending our efforts.

The knowledge of the *omdus operandi* of our remedies is similar to the acquaintance with reagents of the chemist, and the more they are understood the less shall we be guilty of "pouring medicines of which we know little into a body of which we know less."

In the case of the phlegmasiæ the relations of chemistry to therapeutics is similar, although not quite so prominent. The formation of coagulable matter, and its prevention by means of mercurials, &c., offer a series of phenomena known to experience, but not capable of explanation by any chemical laws without the introduction of hypotheses, and in such explanations hypotheses are not admissible ; and the antiphlogistic treatment is received on the same grounds chemically as the use of ferments, &c., in which the knowledge of the faet is made subservient to our purposes, while the *modus operandi* still remains a subject for investigation. That portion of the treatment which relates to the febrile portion of the phenomena and the functional derangements may be referred to the remarks on the treatment of pyrexia.

The neuroses present still greater difficulties to the investigator, both with respect to the chemical changes in the tissues and the nature of the action of remedial agents. The action of the alkaloids, of the oxides and salts of metals, is enveloped in the deepest mystery. Yet, the use of iron, arsenic, silver, iodine, quinia, morphia, strychnia, &c., &c., is so well understood that they are safe and reliable agents in the hands of the educated physician, and disease disappears so continually under their use that any question of cause and effect ceases to be entertained. In all such cases there is a vast field open for research, and it will be only as the chemistry of the subject is cleared up, that our knowledge of the matter will be substantially increased.

The diseases of the chylopoietic viscera offer some of the most striking examples of the application of chemical knowledge to the treatment of disease, and many of the primary and secondary disturbances in indigestion are easily controlled in a manner perfectly in accordance with chemical facts- The use of alkalies in cases of acidity, of pepsine to expedite the process of digestion, of the hyposulphites to prevent fermentation with evolution of gas ; to say nothing of the adaptation of food to the ability and condition of the digestive powers, or of the employment of oxygen, and consequent evacuation of foreign matter, and of the other hygienic means, are striking examples of the advance made by rational medicine beyond the range of empiricism.

Chronic diseases of the liver and of the intestines are only in part amenable to chemical treatment, being mostly influenced by remedies used empirically. The same may be said of diseases of the urinary organs, and still more so of those of respiration and of the skin.

Yet, when we view the cases of spontaneous cure which so frequently come under our observation, and those cases in which "nature" accomplishes a cure in spite of our remedies, we cannot fail to be struck with the fact, that the conditions of health and disease are merely expressions of material changes, and that there are laws governing these changes taking place within the body of which we are yet entirely ignorant; and that it is only as our knowledge of the organism in all its parts and in all its workings becomes perfected, that we shall make real progress in the treatment of its diseases. Experimental chemistry must be our Œdipus in the solution of these problems.—*Amer. Jour. of Med. Science.*

